# Strengthening community engagement for the control of *Aedes*-borne diseases: Insights from an international workshop in Rio de Janeiro, Brazil

**DOI:** 10.1371/journal.pntd.0014832

**Published:** 2026-11-04

**Authors:** Anne Poinsignon, Gabriela Azevedo de Aguiar, Mar Jambou, Jean W. Birba, Irene R. Moshi, Javier Lezaun, Vincent Corbel, Florence Fournet, Marcia F. Lenzi

**Affiliations:** 1 MIVEGEC, University of Montpellier, IRD, CNRS, Montpellier, France; 2 Institutional Relations Office of the President’s Office, Fundação Oswaldo Cruz (Fiocruz), Rio de Janeiro, Brazil; 3 Centre d’Estudis Avançats de Blanes (CEAB-CSIC), Blanes, Spain; 4 Institut de Recherche en Sciences de la Santé (IRSS/CNRST), Ouagadougou, Burkina Faso; 5 Environmental Health and Ecological Sciences Department, Ifakara Health Institute (IHI), Dar es Salaam, Tanzania; 6 Institute for Science, Innovation and Society (InSIS), School of Anthropology and Museum Ethnography, University of Oxford, Oxford, United Kingdom; 7 Instituto Oswaldo Cruz (IOC), Fundação Oswaldo Cruz (Fiocruz), Rio de Janeiro, Brazil; 8 Vice-Presidência de Educação, Informação e Comunicação (VPEIC), Fundação Oswaldo Cruz (Fiocruz), Rio de Janeiro, Brazil; Centers for Disease Control and Prevention, UNITED STATES OF AMERICA

## Abstract

Arboviral diseases transmitted by *Aedes* mosquitoes, including dengue, chikungunya and zika, represent a growing global public health threat, exacerbated by climate change, rapid urbanisation, social inequalities and weak public infrastructures. While technical innovations in vector control continue to expand, their effectiveness and sustainability largely depend on social dynamics, sustained community engagement and local ownership. We report here insights from the International Workshop *Strengthening North-South research cooperation in social sciences for protecting vulnerable populations from* Aedes*-borne diseases*, held in Rio de Janeiro in September 2025. The meeting brought together researchers, public health professionals, and community actors from the Americas, Europe, and Africa. The workshop aimed to explore how social sciences can contribute to the surveillance and control of *Aedes*-borne diseases, particularly in vulnerable settings. Structured around three thematic sessions, the workshop examined (i) how the social sciences can contribute to better health and well-being in a context of global environmental and climatic changes; (ii) what are the factors shaping communities’ perceptions of *Aedes* control strategies, and how can social sciences contribute to appropriation and implementation of those strategies, including *Wolbachia* and sterile insect techniques; and (iii) how citizen science tools and digital technologies support surveillance and engagement in the fight against vector-borne diseases? Across sessions, case studies from Latin America, Africa, and Europe highlighted the central role of trust, locally grounded knowledge, participatory governance, and intersectoral collaborations. Discussions emphasised that community engagement is a core determinant of effective and equitable vector-borne disease control. Citizen science initiatives, participatory surveillance, and co-designed interventions were shown to strengthen data quality, improve acceptability of innovations, and empower communities to address structural determinants of exposure. The workshop concluded that integrating social sciences into One Health approaches is essential to develop adaptive, inclusive, and sustainable responses to arboviral threats in a rapidly and unpredictably changing world.

## Author summary

Dengue, chikungunya and zika are viral diseases transmitted by *Aedes* mosquitoes that affect millions of people worldwide, particularly in urban and socially vulnerable settings. Their spread is influenced not only by mosquitoes, but also by environmental change, material living conditions, social inequalities and how communities interact with public health interventions. Although new technologies for mosquito control are increasingly available, their effectiveness often depends on whether they are understood, accepted and supported by local populations. This article presents key lessons from an international workshop on social sciences held in Rio de Janeiro in 2025. Researchers, public health professionals, and community actors discussed how social scientific research and public engagement can improve the surveillance and control of *Aedes*-borne diseases. The discussions covered community perceptions of mosquito control strategies, the use of citizen science and digital tools for disease surveillance, and field experiences showing how community participation can strengthen prevention efforts. Across different countries and contexts, the workshop highlighted the importance of trust, local knowledge, and collaboration between communities and institutions. These discussions underline that integrating social sciences into One Health approaches can help design more effective, equitable, and sustainable strategies to reduce the burden of arboviral diseases.

## Introduction

Dengue represents a major global health challenge, currently threatening nearly half of the world’s population and causing up to 400 million infections and approximately 40,000 deaths each year [1]. In Brazil alone, 6.3 million cases and over six thousand dengue-related deaths were reported in 2024, disproportionately affecting residents of neglected neighbourhoods, where stagnant water and accumulated waste create ideal conditions for virus transmission [2]. What was once considered a “tropical” problem is now a global threat, with autochthonous outbreaks of dengue being recorded in Europe and the United States in recent years [3,4]. This situation results from a convergence of factors, including environmental and climate change, urbanisation, inadequate basic infrastructure, limited political commitment, and weak community engagement, all of which favour the proliferation of *Aedes aegypti* and *Aedes albopictus* [5].

Community engagement is recognised as a cornerstone of the integrated strategy promoted by the World Health Organization to reduce the global burden of mosquito-borne diseases (MBD) [6]. Evidence shows that participatory approaches, such as community engagement, participatory surveillance, citizen science, co-design and community-led vector control, can significantly reduce dengue transmission [7]. In Cuba, community-based environmental management targeting mosquito breeding sites has led to substantial reductions in *Aedes* infestation levels [8]. In Brazil, community engagement has been instrumental in facilitating the deployment of innovative interventions, including the release of *Wolbachia*-infected mosquitoes, by improving public understanding, acceptance, and local participation [9].

Within a One Health framework, social science–based strategies, including behavioural and social research, stakeholder mapping, and social science–informed community engagement and co-design, can make a substantial contribution to reducing the burden of vector-borne diseases (VBD), as they are adaptable to multiple pathogens, health systems, and geographic contexts [10]. Nevertheless, a persistent challenge remains: how to effectively raise awareness, mobilise communities, and sustain engagement in VBD surveillance and control across diverse educational, linguistic, cultural, and socioeconomic settings.

This challenge was central to the international workshop *Strengthening North-South research cooperation in social sciences for protecting vulnerable populations from* Aedes*-borne diseases*, held on September 22–24, 2025, in Rio de Janeiro, Brazil, organised by the Fundação Oswaldo Cruz (Fiocruz, Brazil) in partnership with the French National Research Institute for Sustainable Development (IRD, France) and supported by the INOVEC Horizon Europe project (Table 1). The workshop brought together researchers from the Americas, Europe, and Africa to share experiences, deepen understanding of citizen motivations and roles in mosquito control programs, and address the specific challenges faced in vulnerable settings, such as favelas and hard-to-reach populations. Its ultimate goal was to develop a framework for an international social science network on arboviruses, fostering collaboration and strengthening innovative and sustainable responses to arboviral diseases, while placing health at the heart of global climate and development justice agendas.

**Table 1:** Examples of initiatives illustrating the contribution of interdisciplinary collaboration, community engagement, and participatory approaches to addressing health and vector-borne disease challenges.

Initiative name	Description	Website
INOVEC	The Horizon Europe INOVEC project (*a research and In*NO*vation Partnership for enhancing the surveillance and control of mosquito* VEC*tors of emerging arboviruses*) aims to strengthen innovative, integrated, and community-based approaches for the surveillance and control of mosquito vectors of emerging arboviruses. Coordinated by the French National Research Institute for Sustainable Development (IRD), INOVEC brings together 26 partners from Europe, Africa, and the Americas, including Brazil, through institutions such as Fiocruz. By fostering interdisciplinary collaboration, capacity building, and knowledge exchange across disciplines and continents, the project aims to develop more effective and sustainable vector surveillance and control solutions adapted to diverse epidemiological and socioeconomic contexts.	https://inovecproject.com/
Redisse IV	The Redisse IV project is a partnership between the Ministries of Health of Angola and Brazil, funded by the World Bank, aimed at updating Angola’s entomological map and strengthening the country’s capacity for vector surveillance. The project combines entomological data collection across diverse ecological settings with the training of local surveillance professionals. Guided by a One Health approach, it addresses a broad range of disease vectors and places particular emphasis on sustainability, capacity building, and collaboration between Angolan and Brazilian institutions. By strengthening local expertise and promoting integrated surveillance, REDISSE IV illustrates how international partnerships can support sustainable national capacities for monitoring and responding to vector-borne diseases.	https://www.fiotec.fiocruz.br/en/news/interview-this-is-a-great-experience-that-should-be-taken-to-other-countries
Humble Brick	The Humble Brick is an interdisciplinary project that brings together experts in entomology, architecture, biosciences, engineering, and urban planning, along with local masons, builders and residents in Tanzania to explore the link between house construction methods and the risk of mosquito-borne diseases. It investigates how traditional earthen bricks could be better used to improve ventilation while reducing entry of *Anopheles* mosquitoes into domestic domiciles. Lessons from this project will lead to locally-driven, climate-adapted and sustainable innovations in building construction and design.	https://gtr.ukri.org/projects?ref=MR%2FZ505742%2F1
Garo Moustik	Garo Moustik is a collaborative serious game designed to raise awareness of *Aedes* mosquito control and promote collective action at the community level. Participants take on the role of residents in a shared neighbourhood and work together to identify and eliminate potential mosquito breeding sites. The game highlights how individual practices are interconnected and how effective vector control requires coordination, communication, and shared responsibility among community members.	.https://umr-astre.cirad.fr/ressources-publications/jeux-serieux/garomoustik
Mosquito Alert	Mosquito Alert is a citizen science platform and mobile app that enables citizens to contribute to mosquito surveillance. Through the app, participants can report mosquito sightings, breeding sites, and mosquito bites, generating georeferenced data that complement traditional surveillance systems. These observations are validated and integrated into scientific and public health workflows, helping researchers and health authorities better understand mosquito distribution, seasonal dynamics, and population trends.	https://www.mosquitoalert.com/en/
EntomoFavela	The EntomoFavela project, coordinated by Fiocruz through the Instituto Oswaldo Cruz (IOC), aims to develop a community-based entomological surveillance approach adapted to the specific realities of urban favelas. The project combines scientific expertise and community engagement to better understand *Aedes aegypti* dynamics and strengthen local capacities for vector surveillance and control. It involves a multidisciplinary partnership between Fiocruz-IOC, Fiopromos, the French National Research Institute for Sustainable Development (IRD), and the French Embassy in Brazil. By training and involving community members as active participants in surveillance activities, EntomoFavela illustrates how long-term partnerships, trust-building, and collaboration between research institutions, communities, and international partners can support innovative and context-sensitive approaches to addressing vector-borne diseases.	https://www.entomofavela.com.br/
Respira Mare	The Respira Maré project, developed by Redes da Maré in collaboration with Fiocruz, aims to assess air quality, heat islands, and their potential impacts on the health of residents of the Complexo da Maré. The project combines environmental monitoring and community engagement, involving local actors in data collection and the production of knowledge to better understand urban environmental health challenges. Respira Maré illustrates how trusted partnerships with communities can support locally driven research and public health actions in vulnerable urban settings.	https://www.redesdamare.org.br/br/info/86/respira-mare
Vacina Maré	The Vacina Maré project, led by Fiocruz in partnership with Redes da Maré and the Municipal Health Secretariat of Rio de Janeiro, aimed to evaluate the impact and effectiveness of COVID-19 vaccination in the Complexo da Maré, one of the largest urban favela complexes in Rio de Janeiro. The initiative combined a large-scale vaccination campaign with scientific research to assess vaccine effectiveness, viral circulation, emerging variants, and post-COVID conditions in a socially vulnerable population. The campaign, conducted in July–August 2021, mobilised local organisations, health professionals, volunteers, and community networks to vaccinate more than 36,000 adult residents within four days.	https://www.vacinamare.org.br/
MOSAIC	The Horizon Europe MOSAIC project (Multi-site application of Open Science in the creAtion of healthy environments Involving local Communities) aims to develop open, participatory, and replicable approaches to better understand and address the links between environmental changes and human health. The project focuses on empowering local communities through the co-production of data and knowledge, enabling them to better identify environmental and health challenges and actively participate in the design of adaptation and mitigation strategies. Coordinated by IRD, MOSAIC brings together 15 partners from seven countries, including research institutions and organisations working across environmental sciences, health, social sciences, and participatory approaches.	https://www.mosaic-planetaryhealth.eu/

To build bridges between scientific knowledge and practical action, between social sciences and public health, and between local and global levels of action, the workshop was structured around three thematic sessions, each addressing a key question: (1) how the social sciences can contribute to improving health and well-being in a rapidly changing environment? (2) What are the factors shaping communities’ perceptions of *Aedes* control strategies, and how can social sciences contribute to appropriation and implementation of those strategies, including *Wolbachia* and sterile insect techniques (SIT)? (3) How can citizen science tools and digital technologies support surveillance and engagement in the fight against VBDs? This program also included keynote lectures and a roundtable on “*Challenges and opportunities in community engagement for vector-borne disease control*.” The conference gathered over 60 in-person participants including researchers, public policy makers, and representatives of national and international organisations, with an additional 37 remote participants.

## Welcoming addresses

The workshop opened with remarks from institutional representatives of Fiocruz, the French Embassy in Brazil, IRD Brazil, and the French Agency for Research on Emerging Infectious Diseases (ANRS-MIE), who emphasised the importance of strengthening international collaboration to address the growing burden of arboviral diseases in a context of climate and social change. The opening keynote introduced broader perspectives on global health in Brazil and highlighted the intersection between geopolitics, climate change, and social inequalities, and the growing burden of arboviral diseases.

## Keynote lectures

Three keynote lectures then set the scientific and epidemiological context for the sessions that followed, addressing the interconnected public health challenges posed by mosquito-borne threats. Together, these presentations highlighted current challenges and strategic approaches in the prevention and control of VBDs.

An updated epidemiological overview of arboviruses in the Americas highlighted concerning regional trends and situated the workshop discussions within the context of an unprecedented disease burden. The year 2024 represented the most severe dengue epidemic in the continent’s recorded history, with more than 13 million probable cases [11]. By mid-2025, Brazil had reported over 3.6 million cases, with all four virus serotypes circulating simultaneously. A rising incidence of chikungunya over the past five years was also pointed out, along with more than 20,000 laboratory-confirmed zika cases in 2025, and a notable increase in Oropouche virus transmission, particularly in Brazil, Cuba, and Panamá, representing a 27% rise from 2024 [12]. Yellow fever outbreaks remained concentrated in South America, with 331 confirmed cases and a high case fatality rate of 41.7%. Climate change was identified as a major factor reshaping the epidemiology of MBDs [13]. The recent expansion of *Aedes aegypti* into southern Brazil and northern Argentina, areas previously considered less vulnerable, stands as clear evidence of climate-driven shifts in vector distribution and disease dynamics.

International health cooperation in the context of Brazil–Africa partnerships in vector surveillance was then examined. Drawing on Fiocruz’s cooperation model, developed in the 2000s and grounded in principles of ownership, alignment, harmonisation, results-orientation, and mutual responsibility, an alternative to traditional North–South cooperation paradigms was proposed. This approach was situated within the long-standing debate between vertical disease-control programs, focussed on fighting specific diseases, and horizontal strategies aimed at strengthening health systems. This model was illustrated by the 2025 update of Angola’s entomological map, conducted within the Redisse IV project (Table 1). Initially conceived as a consultancy initiative addressing major endemic diseases, including malaria, dengue, and yellow fever, the project evolved into a broader program combining training, surveillance, and capacity building. Activities included hybrid training on vector morphology and data management, followed by extensive fieldwork across eight provinces involving 200 students. This “diagonal approach” used disease-specific resources (vertical resources) to strengthen broader health system capacity, engaging surveillance teams and communities. The persistent challenges related to cultural and communication barriers, bureaucratic rigidity, institutional constraints, and long-term project sustainability underscored the message that “the biggest disease is poverty.”

Community mobilisation approaches for the evaluation and control of *Aedes aegypti* in Brazil were presented. Effective vector control was shown to be critically dependent on community mobilisation, as more than 90% of *Aedes* breeding sites are located within households and peridomestic environments, spaces that public health systems can only reach with community consent and participation. It was also noted that commonly used entomological indices, such as the House Index, exhibit low sensitivity in predicting dengue outbreaks, reinforcing the need for integrated, multidimensional monitoring systems combining entomological, epidemiological, climatic and social indicators. Drawing on experiences from Fortaleza, a city with longstanding co-circulation of dengue, chikungunya, and zika [14], persistent challenges in urban vector control were identified, including cryptic breeding sites, inadequate waste management and sanitation, and marked socioeconomic disparities. In some areas, such as urban favelas, organised crime has directly obstructed vector-control activities, with evidence suggesting that higher homicide rates correlate with increased dengue incidence during epidemics [15]. In response to these complex and interconnected challenges, the Eco-Bio-Social framework was presented as a transdisciplinary approach to culturally adapted health education and vector control [16]. Community mobilisation was ultimately framed as a mechanism to strengthen local ownership, foster behavioural change, strengthen trust in authorities, and improve the sustainability of surveillance and control activities.

## Session 1. Global changes and impact on communities: How the social sciences can contribute to better health and well-being?

This session examined how global environmental and climatic changes translate into concrete health challenges at the community level, and explored how perspectives from the social sciences can contribute to more effective, equitable, and context-sensitive responses. Across the discussions, three interconnected themes emerged: understanding health risks as socio-ecological processes, designing interventions that account for local contexts and capacities, and strengthening community engagement in the design of health interventions as a mechanism for improving health governance.

The need to move beyond linear understandings of environmental change and health outcomes was addressed. Several contributions highlighted that the distribution of VBDs is shaped by complex interactions between climatic, ecological, spatial, and socioeconomic factors operating across different scales. Examples presented during the session illustrated how interventions generally considered beneficial from an environmental perspective may produce unintended public health effects if vector ecology is not adequately considered. In particular, urban greening initiatives, despite their recognised environmental and social benefits [17], may under certain conditions increase opportunities for vector breeding or alter patterns of human exposure to disease vectors [18,19]. These discussions emphasised that urban and environmental policies should incorporate epidemiological and ecological evidence from the early stages of planning and implementation.

A second theme focussed on the importance of developing interventions that respond to local realities rather than relying on standardised solutions. Examples discussed during the session showed how approaches grounded in local production systems, housing practices, and territorial conditions can contribute to reducing disease exposure while remaining feasible and acceptable for local populations [20]. Initiatives such as the *Humble Bricks* project (Table 1) emphasised co-design processes involving residents and local actors throughout the development and implementation of interventions. More broadly, the discussions highlighted that local capacities should not be treated simply as implementation resources but must be recognised as a source of knowledge and innovation capable of improving intervention effectiveness and sustainability.

The contribution of participation and community engagement to health adaptation in contexts of environmental change was also discussed. Climate change was described as affecting health both directly, through changes in the ecology and distribution of vectors and pathogens, and indirectly, through processes such as population displacement, economic vulnerability, and increased pressure on health systems. In this context, several contributions stressed the importance of intersectoral approaches connecting health, environmental, and urban policies, together with mechanisms that strengthen community participation and collective action. Participatory surveillance initiatives discussed during the session provided concrete examples of how local mobilisation can contribute to territorial health governance. Field experiences, including the MOSAIC, EntomoFavela, and Vacina Mare [21] projects (Table 1), illustrated how community engagement can support the design, implementation, and sustainability of vector control and public health programs. In the context of dengue prevention, these initiatives contributed not only to the identification of vector breeding sites but also to a broader understanding of the environmental and social determinants of exposure, including sanitation conditions, waste accumulation, water storage practices, and patterns of urban infrastructure. Participatory approaches, including stakeholder mapping, community workshops, participatory cartography, territorial diagnosis, and the co-development of educational and communication tools, enabled residents to help identify local priorities and ensure that health messages were culturally appropriate and tailored to the socio-linguistic realities of the territory. In cross-border and socially heterogeneous contexts, these approaches helped adapt surveillance systems to complex local social organisation, communication practices, and environmental realities. By involving residents directly in the collection of entomological and environmental information, they generated locally relevant evidence while fostering dialogue between communities, researchers, and public institutions. Beyond strengthening surveillance, these initiatives promoted community ownership, collective action to improve local sanitation, and advocacy for better public services. The discussions also highlighted that sustained collaboration between research institutions and community organisations is essential in contexts characterised by social vulnerability and structural inequalities.

Overall, the session suggested that responding to climate- and environment-related health challenges requires moving beyond narrowly technical approaches. Social science perspectives contribute by revealing how environmental risks are produced and experienced in specific social and territorial contexts and by supporting interventions that combine scientific evidence with local knowledge, participation, and long-term institutional engagement.

## Session 2. Environmental management, SIT, and *Wolbachia*-carrying mosquitoes: Community perceptions and social scientific contributions to their appropriation and implementation

The second session focussed on the pivotal role of community engagement and social science in supporting the successful implementation of emerging noninsecticide-based approaches to mosquito control, developed in response to increasing insecticide resistance and the recognised limitations of indoor-focussed interventions. These strategies, including *Wolbachia*, the SIT, and larval source management (LSM), primarily target outdoor breeding sites and mosquito populations at different stages of the mosquito life cycle to reduce vector populations and transmission risk. Across the interventions, a common conclusion emerged: technological effectiveness alone does not ensure successful implementation. Long-term impact depends on how interventions are understood, accepted, and integrated within local social contexts and institutional capacities.

Experiences presented during the session, including the deployment of *Wolbachia*-infected mosquitoes in Rio de Janeiro, Brazil [9], and in Medellín, Colombia [22], as well as the deployment of the SIT in Cuba [23], illustrated that community engagement should not be viewed merely as a communication strategy aimed at convincing communities to accept a technology. Rather, it should create opportunities for informed participation and enable interventions to take into account local expectations, concerns, social organisation, and cultural and economic contexts. Prior to the releases, early stakeholder mapping, community profiling, dialogue with local representatives, and the establishment of community reference groups have provided mechanisms for understanding local contexts, including the social organisation of territories, existing communication networks, institutional relationships, and the actors shaping participation and decision-making. These experiences also demonstrated the value of integrating social scientists, geographers, communication specialists, and public health expertise alongside entomological and epidemiological knowledge. Such interdisciplinary approaches helped identify locally appropriate strategies and adapt interventions to the realities of the communities.

Importantly, these experiences showed that community engagement needed to be sustained throughout all phases of intervention, rather than being limited to the period preceding implementation. Engagement began with listening to communities through a range of consultative spaces, including awareness campaigns, in-person meetings, community events, and online platforms. These provided opportunities for residents to access information on the intervention, ask questions, express concerns, and contribute to shaping its implementation. During implementation, communication channels involving community leaders, health workers, schools, local authorities, and radio and social media were identified as important entry points for fostering participation and ensuring transparency. Issue-management systems were also implemented in the *Wolbachia* release programs to identify and respond to concerns, misconceptions, or unforeseen problems, and to adapt intervention strategies, including communication, when needed. In addition, periodic surveys assessing community awareness and acceptability enabled programs to monitor perceptions and adapt their strategies accordingly. In Cuba, for instance, such monitoring documented strong political support and high community acceptance of the SIT (67.9%), particularly among women, people under 60, and individuals of European descent [23]. Together, these experiences demonstrate that early engagement, sustained dialogue, and mechanisms for monitoring community perceptions can contribute to program legitimacy and facilitate the uptake of new vector control interventions.

Despite these positive experiences, the session also highlighted challenges that can limit the successful implementation of vector control interventions. Two complementary limitations emerged: inadequate community engagement and broader structural and institutional constraints. The first was illustrated by a case study from a dengue-endemic setting in Africa, where rumours and misinformation, exacerbated by the exclusion of communities from the design and implementation of government-led interventions, undermined trust and program effectiveness. This experience showed that communication introduced only after decisions have been made may be insufficient to address concerns or restore confidence. Meaningful participation therefore needs to be embedded from the outset, with mechanisms allowing communities to raise concerns and programs to respond and adapt throughout implementation.

A second example, from the Kilombero and Handeni districts of Tanzania, highlighted the challenges encountered in implementing LSM for malaria control. Despite strong political commitment and recognised public health potential, implementation was constrained by insufficient funding, fragmented coordination across sectors, unclear governance arrangements, and limited operational capacity. In particular, coordination between the malaria control programme and the neglected tropical diseases programme within the Ministry of Health was limited. Limited community engagement during the early phases, and insufficient access to training and operational resources, including bio-larvicides (biological insecticides used to control mosquito larvae in breeding sites, such as *Bacillus thuringiensis subsp. israelensis* (Bti) and *Lysinibacillus sphaericus*), further complicated implementation.

## Session 3. Citizen-based science tools and technologies: how can these new approaches be used in the fight against vector-borne diseases?

This session explored how citizen-based approaches, including citizen science and participatory technologies, can complement conventional vector surveillance and control strategies. Citizen science was discussed as a promising framework for fostering more inclusive and meaningful engagement, based on democratic participation and the co-production of knowledge by citizens and researchers [24]. Across the discussions, a consistent theme emerged: citizens are not only recipients of public health interventions but can become active contributors to surveillance, prevention, and collective decision-making. The examples presented illustrated how participatory tools, including educational games, mobile applications, can generate actionable data, strengthen community ownership, and support more adaptive responses to the prevention and control of *Aedes*-borne diseases.

Participatory tools can play an important role in supporting learning and behavioural change. The educational approach presented during the session aimed to foster active learning and collective engagement rather than simply raising awareness. One example was the development of the serious game *GaroMoustik* (Table 1), initially designed for the context of Réunion Island as a collaborative board game designed to promote collective action for the control of *Aedes* mosquito vectors. By simulating neighbourhood-level decision-making, the game encourages participants to coordinate actions to reduce mosquito breeding sites and limit disease transmission. By recreating the practical challenges of collective action, including coordination, shared responsibility, and risk management, it transforms theoretical knowledge into practical action and fosters the adoption of effective behaviours. Its adaptability across different territorial contexts and its use by public health actors illustrate its potential as a tool for strengthening community mobilisation and preventive practices.

Citizen-generated data can also serve as a valuable resource for surveillance and proactive public health action. The *Mosquito Alert* citizen science platform (Table 1) presented during the session demonstrated how mobile technologies, participatory reporting, and artificial intelligence-based image validation can expand the reach and responsiveness of entomological surveillance systems [25]. This approach enables residents to report mosquito observations and environmental conditions, generating near real-time data that support surveillance, early warning, and public health decision-making. Although initially developed to strengthen the early detection of invasive *Aedes* species in Spain, such as the tiger mosquito *Ae. albopictus*, discussions highlighted that such platforms have value in endemic settings where vectors are already established. For example, it has been successfully adapted for use in Uruguay following the major dengue epidemic of 2023–2024, prompting coordinated national responses. In such settings, citizen-generated data can support the monitoring of mosquito abundance and spatial distribution, identify local transmission hotspots, guide targeted vector control interventions, and sustain public awareness and community engagement over time.

The discussions also highlighted that the long-term success of participatory surveillance depends on its integration into existing public health systems and sustained institutional support. The effectiveness of citizen-based technologies depends not only on technical performance but also on the social and institutional arrangements that support their implementation. Stakeholder mapping approaches illustrated how actors, including government agencies, academic institutions, social organisations, media, religious groups, community groups, and international partners, play different roles in shaping participation, ownership and sustainability. As presented here, the stakeholder-mapping approach developed to support the pilot implementations of *Mosquito Alert* in endemic settings such as Tanzania and Burkina Faso revealed that variations across implementation settings require context-sensitive engagement strategies. These strategies are essential for building trust, coordinating actors across sectors, and establishing mechanisms for sustained collaboration.

Overall, the session demonstrated that citizen-based science tools can contribute to vector control not only by producing additional data but also by reshaping relationships between communities, researchers, and public institutions. Their added value lies in their capacity to combine surveillance, education, and participation, thereby strengthening both preparedness and the long-term governance of VBD prevention.

## Round table: Challenges and opportunities in community engagement for vector-borne disease control

The workshop roundtable emphasised that, despite advances in vector surveillance technologies and control strategies, the sustainable control of arboviral diseases remains highly dependent on community participation and social engagement [26]. Vulnerability to arboviral diseases is strongly shaped by living and environmental conditions, access to public services, and underlying structural inequalities, making community engagement a central but complex pillar of health promotion programs [27]. Without the active involvement of affected populations, interventions rarely achieve long-term effectiveness. This challenge is shared by countries in both the Global South and the Global North, albeit in highly diverse socio-cultural, political, and environmental contexts [28]. The roundtable aimed to create a space for collective reflection on how community participation and engagement are understood, encouraged, and implemented in VBD surveillance and control. Participants were invited to share experiences, challenges, and critical perspectives on community participation and/or engagement arising from work in diverse territories. The roundtable adopted a participatory discussion format and lasted approximately one hour, bringing together approximately 50 participants, including researchers from different countries and continents with varied levels of experience, community agents from informal settlements, and university students. Many key points were raised about how academia and research institutions encourage or facilitate public participation in surveillance and/or control projects for *Aedes* mosquitoes.

### Defining participation and engagement

One of the first themes to emerge was the distinction between participation and engagement. Participation was often described as passive or individual, such as allowing researchers to install mosquito traps, or agreeing to undergo health tests, whereas engagement implies a deeper, collaborative process where communities are involved from the earliest stages of a project, including the definition of objectives, the identification of priorities, and shared responsibility in planning, implementation, and monitoring. Engagement thus recognises communities as partners rather than research subjects.

### Barriers to effective engagement

Trust emerged as a central determinant of engagement. Entomological agents and community-based actors were identified as key intermediaries given their embeddedness in local contexts and their intimate understanding of community realities, positioning them as important bridges between public health systems and residents. However, the discussion also highlighted the need for adequate training and awareness-raising among these professionals regarding their role not only as data collectors, but as agents of dialogue and engagement. Researchers, by contrast, must actively build trust when entering a territory, as they lack pre-existing relationships with residents. Institutional credibility was repeatedly cited as a decisive factor influencing community willingness to engage. Trust in the institution leading a project can significantly ease collaboration and acceptance. This has been illustrated by the example of Fiocruz in Brazil, which has established a long-standing, trusted partnership with communities in the Complexo da Maré favelas, building a high level of trust with residents over many years. This sustained relationship has enabled the successful implementation of numerous public health interventions and research projects, including Respira Maré, Vacina Maré, and EntomoFavela (Table 1). This example illustrates how long-term community engagement and trust can facilitate both research and public health interventions in underserved urban settings.

A recurring concern was participation fatigue among communities frequently solicited by research projects [29], often linked to confusion about the distinction between research activities and public health interventions. When projects are perceived to carry a promise of immediate solutions but result mainly in data collection, disappointment and disengagement often follow. Clear communication and transparency regarding project objectives and expected outcomes were therefore identified as essential.

In some contexts, punitive measures, such as fines for household breeding sites [30], can undermine cooperation, particularly in vulnerable settings where residents lack the means to reduce environmental risks, thus fostering blame rather than cooperation. Instead, supportive approaches that empower communities to manage breeding sites, especially in areas lacking resources, are more effective. The roundtable further emphasised the limitations of approaches that focus solely on individual or household responsibility while neglecting structural determinants such as poor sanitation, inadequate infrastructure, and insufficient public services.

### Practical strategies for engagement

Popular entomological surveillance, led by trained community members, was highlighted as a powerful mechanism for integrating territorial knowledge into research and action [31]. Community members bring lived experience, social networks, and contextual understanding that researchers alone cannot access. By valuing local knowledge and lived experience, these approaches enhance data reliability and bridge the gap between academic research and community needs.

However, the selection of community agents remains a sensitive issue. Academic teams may implicitly favour individuals with higher levels of formal education or prior research experience, excluding others deemed “inexperienced”. Participants challenged this bias, noting that formal credentials do not necessarily predict commitment, leadership, or capacity for engagement. Such practices risk reinforcing existing inequalities that participatory approaches seek to overcome.

The roundtable underscored the importance of cultural sensitivity and recognition of existing knowledge systems, including ancestral and traditional practices. Researchers must begin by understanding the territories they aim to serve. This includes assessing local health priorities, mapping existing networks, and also local associations or NGOs that are very important for facilitating project acceptability and implementation (e.g., *Redes da Maré* NGO in the favela Maré in Rio de Janeiro), and the impacts of climate change. Projects should be designed in collaboration with community leaders, ensuring they reflect local realities and priorities [32]. In some communities, hierarchical social structures mean that participation is mediated by local leaders, which can both facilitate and constrain engagement. Participants also noted that in highly vulnerable settings, particularly those affected by climate change, violence, or food insecurity, the control of arboviral disease may not be perceived as an immediate priority. In such contexts, researchers must first understand the broader landscape of needs and ongoing struggles before proposing engagement strategies.

### Concluding remarks

Community participation and social engagement emerged as essential components of sustainable control of *Aedes* mosquitoes. The discussion highlighted that effective interventions must be grounded in local realities and built on trust, dialogue, and shared responsibility among communities, public authorities, and other stakeholders.

## Conclusion

This international workshop highlighted the central role of social sciences in understanding and addressing the complex social, environmental, and institutional dimensions of arbovirus prevention and control. Across diverse geographic, cultural, and epidemiological contexts, participants consistently highlighted that sustainable mosquito control cannot rely solely on technological solutions. Social inequalities, living conditions, governance structures, and trust relationships fundamentally shape both exposure to arboviral diseases and the effectiveness of control strategies.

The experiences presented during the workshop demonstrate that meaningful community engagement, understood as a collaborative and empowering process rather than simple participation, is a prerequisite for durable vector surveillance and control. Citizen science tools, community-based entomological surveillance, participatory communication strategies, and co-designed interventions emerged as powerful approaches to bridge scientific knowledge and everyday realities, particularly in vulnerable settings such as informal urban settlements, border regions, rural and Indigenous communities. Importantly, the discussions also revealed common challenges, including participation fatigue, institutional mistrust, security constraints, and the risk of placing disproportionate responsibility on individuals while structural determinants remain unaddressed. These challenges call for long-term commitments from public authorities, sustained investment in basic services, and closer integration between research, public health action and community priorities. Overall, the workshop reaffirmed the relevance of a One Health perspective that fully integrates social sciences as a central pillar of arbovirus prevention and control. Strengthening North–South and South–South research cooperation, fostering interdisciplinary collaboration, and recognising communities as key stakeholders are essential to build resilient, equitable and context-sensitive responses to the growing global burden of VBDs. This has been the mission of the Horizon Europe-funded INOVEC project, which has built bridges across disciplines, sectors, and continents to foster collaboration and develop more effective, innovative, and sustainable solutions for vector surveillance and control in Europe and beyond. The creation of an international social science network on arboviruses represents a concrete step toward this goal, aiming to sustain dialogue, share methodologies, and support collective action in the face of evolving arboviral threats.

## Abbreviations

Fiocruz: Fundação Oswaldo Cruz
IRD: French National Research Institute for Sustainable Development
LSM: larval source management
MBD: mosquito-borne diseases
SIT: sterile insect technique
VBD: vector-borne diseases.
